# The Pretreatment of Xiaoqinglong Decoction Alleviates Inflammation and Oxidative Damage and Up-Regulates Angiotensin-Converting Enzyme 2 in Lipopolysaccharide-Induced Septic Acute Lung Injury Rats

**DOI:** 10.1155/2022/2421198

**Published:** 2022-09-19

**Authors:** Jing-Chao Su, Chen Cheng, Cai-Yun Wang, Yi Zhang, Ya-Ting He, Zi-Wei Guo, Xin-Yue Liu, Wen-Mei Liu, Yu-Jie Zhang, Shu-Wen Xu, Xin-Yue Zhang, Zi-Bing Liu, Xin-Fang Zhang

**Affiliations:** ^1^College of Integrated Chinese and Western Medicine, Anhui University of Chinese Medicine, Hefei 230038, Anhui, China; ^2^Graduate School, Anhui University of Chinese Medicine, Hefei 230038, Anhui, China; ^3^Institute of Acu-Moxibustion and Meridian, Anhui University of Chinese Medicine, Hefei 230038, Anhui, China

## Abstract

Xiaoqinglong decoction (XQLD), a classic prescription of Traditional Chinese Medicine, has already been used clinically to cure acute lung injury (ALI), but its mechanism remains unclear. This subject aimed to explore the preventive role of XQLD in septic ALI rats besides its effects on angiotensin-converting enzyme (ACE)2 and its downstream factors. After, respectively, administrated with different concentrations of XQLD (6.25 g/kg/d, 12.5 g/kg/d, 25 g/kg/d) for 5 days and dexamethasone (DEX, 1 mg/kg) for 0.5 h, the rat models of ALI were established by intraperitoneal injection of lipopolysaccharide (LPS, 5 mg/kg) for 24 h. All rats were evaluated by lung function test, arterial blood gas analysis, morphological observation, lung wet/dry (W/*D*) ratio, and the lung injury score. The levels of malonaldehyde (MDA), superoxide dismutase (SOD), interleukin (IL)-1*β*, tumor necrosis factor (TNF)-*α*, and angiotensin (Ang) (1–7) in the lung were measured through biochemical and ELISA kits. The expressions of angiotensin-converting enzyme (ACE)2, mitochondrial assembly receptor (MasR), and nuclear factor (NF)-*κ*B in lung tissue were detected by qRT-PCR and western blotting. Positive reaction cells of MasR were observed by immunohistochemistry. The results show that XQLD significantly ameliorated septic lung injury including edema and hemorrhage, as well as improved pulmonary function and arterial blood gas. Furthermore, XQLD markedly decreased the levels of IL-1*β*, TNF-*α*, MDA, and NF-*κ*B while increased the levels of SOD, Ang (1–7), ACE2, and MasR in septic ALI rats. Pearson correlation showed that the expressions of ACE2 were inversely related to IL-1*β*, TNF-*α*, MDA, and NF-*κ*B and positively correlated with SOD contents. Our data indicated that XQLD pretreatment alleviated inflammation and oxidative damage in septic ALI rats, which might be related to the up-regulation of ACE2-Ang (1–7)-MasR axis and inhibition of the NF-*κ*B pathway.

## 1. Introduction

Sepsis is a heterogeneous syndrome caused by a disordered host response to infection [1]. It is characterized by overwhelming inflammatory responses and oxidative stress, which eventually lead to lethal multiple organ dysfunction [2, 3]. Furthermore, the lung is a target organ vulnerable to sepsis, among which acute respiratory distress syndrome (ARDS) frequently occurs [4]. As a grievous subtype of acute lung injury (ALI), ARDS is the leading factor of death from sepsis [5]. However, there is no effective therapy for septic ALI that exists today.

XQLD, which is a soup made from a mixture of eight natural herbs (Table 1), was first put forward in the book “Shanghanlun” written by a doctor named Zhang Zhongjing of the Han Dynasty in China. After nearly two thousand years of clinical application, it has been proven to be effective and safe at a certain dose, and is still used to extensively treat respiratory diseases to the day, such as cold, allergic rhinitis, acute bronchitis, bronchial asthma, and chronic obstructive pulmonary disease [6]. In the septic ALI rat models induced by intravenous tail injection of LPS, the pretreatment of XQLD significantly alleviated the edema and hemorrhage, and markedly decreased the expression of the Toll-like receptor 4 mRNA in the lung tissue [7]. In addition, XQLD reduced LPS-induced acute injury, inflammatory response, and apoptosis in ALI mice by regulating arachidonic acid metabolism [8]. In a murine model of ovalbumin-induced allergic asthma, XQLD reduced inflammatory cell infiltration and symptoms of airway remodeling [9]. Moreover, studies have shown that XQLD could reduce LPS-induced airway inflammatory cell infiltration [10, 11], and repress the expression of inducible nitric oxide synthase and matrix metalloproteinase-9 in lung tissue via inhibiting the NF-*κ*B pathway [10]. All these demonstrate that XQLD is a potential treatment for lung inflammation.

ACE2 is considered to involve in the regulation of inflammation, oxidative stress, proliferation, and fibrosis in pulmonary diseases, including chronic obstructive pulmonary disease, asthma, pulmonary fibrosis, and ALI [12]. Related studies have reported that the disorder of tissue inherent renin-angiotensin system (RAS) partakes in the nosogenesis of ALI [13]. As a component of the RAS, ACE2 decreases the expression of AngII via impelling the transition of AngII to Ang (1–7). Conversely, Ang (1–7) antagonizes the actions of AngII, thus attenuating the vasoconstriction, apoptosis, inflammatory, and oxidative stress through activating MasR [14, 15], and plays a pivotal part in the origination and development of lung injury [16]. It is reported that the NF-*κ*B activated by AngII promotes the transcription of inflammatory cytokines and aggravates the progression of lung injury [17, 18]; furthermore, the ACE2-Ang (1–7)-Mas axis could alleviate ALI stimulated by LPS by inhibiting the extracellular signal-regulated kinase (ERK) 1/2 and the NF-*κ*B pathway [19].

Therefore, we aimed to investigate the preventive effect of the XQLD in LPS-induced septic ALI rats, and explore its possible anti-inflammatory and antioxidant mechanisms based on the ACE2-Ang (1–7)-Mas axis and the NF-*κ*B pathway.

## 2. Materials and Methods

### 2.1. Reagents

Lipopolysaccharide (No. BS904) was purchased from Saiguo Biotech Co., Ltd (Guangzhou, China). Dexamethasone (No. D8040) was obtained from Solarbio Science Technology Co., Ltd (Beijing, China). The ELISA kits of IL-1*β*, TNF-*α*, and Ang (1–7) (No. JYM0419Ra; JYM0635Ra; JYM0827Ra) were produced by Jiyinmei Biotech Co., Ltd (Wuhan, China). The MDA and SOD kits (No. A003-1; A001-1) were purchased from Jiancheng Bioengineering Institute (Nanjing, China). The rabbit anti-ACE2 antibody (No. bs-1004R) was synthesized by Bioss (Boston, USA). The rabbit anti-MAS1L antibody (No. 20080-1-AP) and *β*-actin antibody (No. 66009-I-IG) were purchased from Proteintech Group (Chicago, USA). The rabbit anti-NF-*κ*B p65 antibody (No. ab16502) was provided by Abcam (Cambridge, UK). The Goat anti-rabbit IgG (No. ZB-2301) was provided by Zsbio Science Technology Co., Ltd (Beijing, China). The horseradish peroxidase-conjugated secondary antibody (No. PV-6000) was provided by Zsbio Science Technology Co., Ltd (Beijing, China).

### 2.2. Plant Materials and the XQLD Production

Eight species of air-dried medicinal herbs used in XQLD (Table 1) were purchased from the First Affiliated Hospital of Anhui University of Chinese Medicine (Anhui, China). After being immersed with purified water, the materials of herbal formula were decocted for reflux extraction, and the hydrous extract with a concentration of 0.5 grams of raw material per milliliter was acquired. The decoction was enriched at 30°C by rotary evaporation to different concentrations and stored at 4°C.

### 2.3. Preliminary Chemical Analysis of XQLD

XQLD was quantitatively analyzed by ultra-high performance liquid chromatography/tandem mass spectrometry (UPLC-MS/MS) and gas chromatography-mass spectrometry (GC-MS) [20]. Then, 2.0 g of sample was weighed, mixed with 50 ml of methanol, and centrifuged at 10000 rpm for 10 minutes. The supernatant was diluted 100 times and injected the sample into the chromatograph. The reference standards were diluted and passed through a 0.22 *µ*m filter membrane. During the process of UPLC-MS/MS (Shimadzu LC-MS-MS-8050), a Waters ACQUITY UPLC C18 column (100 mm × 2.1 mm, 1.7 *µ*m) was used to separate paeoniflorin, glycyrrhizic acid, and schizandrin (all three were 100 ng/ml). The column temperature was maintained at 35°C. Multi-reaction monitoring in positive and negative ion analysis mode includes nitrogen 10.0 L/min, air 10.0 L/min, argon 270 kPa, atomization gas 3.0 L/min, the interface temperature 200°C, the residence time 150 ms, the delay time 3 ms, and the interface voltage 1.0 kv. In GC-MS (Shimadzu GC-MS-2010), cinnamaldehyde and methyl eugenol (both were 50 *µ*g/ml) were separated on a 230°C DB-17MS column (30m × 0.25 mm, 0.25 *µ*m) without shunt. One µL of the sample was injected at a flow rate of 1.0 mL/min, followed by temperature programming. The MS conditions are as follows: ion source temperature was 230°C, interface temperature was 280°C, and delay time was 6 minutes.

### 2.4. Experimental Animals and Groups

Male Sprague-Dawley rats (200 ± 10g) were purchased from the Animal Center of Anhui Medical University [license No: SCXK (Wan) 2017–001], housed at room temperature (21 ± 1°C) with 12h light/dark cycles, and were allowed to freely eat and drink water. All rats were randomly divided into six groups with eight rats in each: Control, ALI, XQLD low-dose (XQLD-L), XQLD medium dose (XQLD-M), XQLD high dose (XQLD-H), and dexamethasone (DEX). XQLD at 6.25 g/kg/d was the minimum dose (XQLD-L) which corresponds to 1 g/kg/d (60 g/60 kg/d as dried herbal amount) for human dose [10], while the XQLD-M and XQLD-H groups were gavaged with 12.5 g/kg/d and 25 g/kg/d, respectively. After oral administration twice daily (at 9 am and 6 pm) for five days, rats except the control group were injected intraperitoneally with LPS (5 mg/kg). Notably, 30 minutes before the LPS challenge, the DEX rats were intraperitoneally injected with dexamethasone (1 mg/kg). All procedures were approved by the Anhui University of Chinese Medicine Ethics Committee.

### 2.5. Measurement of Lung Function

The rats were anesthetized 24 hours after the LPS challenge by intraperitoneal injection of sodium pentobarbital (50 mg/kg). After endotracheal intubation, rats were placed supine in the plethysmograph, and the lung function of rats was evaluated with AniRes 2005 Lung Function System (Bestlab Company, Beijing, China) as the following parameters: forced vital capacity (FVC), the forced expiratory volume (FEV) in 0.1 s (FEV0.1), 0.3 s (FEV0.3), and the ratio of FEV0.1 or FEV0.3 to FVC (FEV0.1/FVC, FEV0.3/FVC).

### 2.6. Arterial Blood Gas Analysis

To evaluate the arterial blood gas of the rats, blood was collected from the abdominal aorta at 24 h after LPS administration. The blood gas analyzer was used to measure the oxygen partial pressure (PaO_2_), carbon dioxide partial pressure (PaCO_2_), pH value, and blood oxygen saturation (O_2_Sat).

### 2.7. Lung Wet/Dry (W/*D*) Ratio

The left lower lobe of the lung was collected to assess pulmonary edema. The weight of fresh left lung tissue was weighed with an electronic balance as wet weight, then the dry weight was obtained after 36 hours in a thermostatic oven at 60°C. The wet/dry (W/*D*) was reckoned.

### 2.8. Histopathology

The upper lobe tissue of the right lung was soaked in 10% formalin for 24 h and embedded after dehydration. The 4-*µ*m thick sections were cut and dyed with hematoxylin and eosin (H&E) for histological examination. The histopathologic score was evaluated following the method described by Yu et al. [21]. Briefly, the degree was mainly assessed by observing four items of hemorrhage, edema, inflammatory infiltration, and proliferation in microscopic damages. According to the order of severity, each scoring item was divided into 0, 1, 2, 3, and 4. The sum of all four items indicates the degree of lung injury. Single-blind scoring was performed by one pathologist according to the severity of the injury under an optical microscope (Olympus, Japan).

### 2.9. Enzyme-Linked Immune Sorbent Assay (ELISA)

After trachea intubation, the right lung of rats was ligated, normal saline was used to lavage the upper lobe of the left lung three times, and the bronchoalveolar lavage fluid (BALF) was collected. The supernatants of BALF and the middle lobe of the right lung homogenate were separated by centrifugation at 3000 rpm for 15 minutes at 4°C. The IL-1*β*, TNF-*α*, and Ang (1–7) contents of BALF and lung tissue in septic ALI rats were determined by ELISA kits.

### 2.10. Measurement of Oxidative Stress

MDA is considered a lipid peroxidation marker, especially in the lungs. SOD protects the lung tissue from oxidative damage by scavenging reactive oxygen species (ROS). The levels of MDA and SOD in BALF and lung tissue in septic ALI rats were assessed according to the manufacturer's instructions for the chemical detective kits.

### 2.11. qRT-PCR

The mRNA expressions of ACE2, MasR, and NF-*κ*B in the lung tissues of rats were examined by quantitative real-time PCR (qRT-PCR). The primer sequences are shown in Table 2, designed by Sangon Biotech (Shanghai), and the reaction system includes 2×SYBR Green mixture (5 *µ*l), forward primer (10 *µ*M, 1 *µ*l), reverse primer (10 *µ*M, 1 *µ*l), cDNA (1 *µ*l), and RNase-free water (2 *µ*l). Samples were incubated for an initial denaturation at 95°C for 1 min, followed by 40 cycles at 95°C for 20 s and 60°C for 1 min. The *β*-actin served as the internal parameter, and the 2^-△△Ct^ value of relative expression of the target gene in each sample was calculated accordingly.

### 2.12. Western Blot

The total proteins of fresh lung tissues in rats were extracted by radio immunoprecipitation assay lysis fluid (Beyotime, No. P0013 B). Then the samples were centrifuged at 12,000 rpm at 4°C for 10 min. After the separation of SDS-polyacrylamide gel electrophoresis buffer, they were transferred to a polyvinylidene fluoride (PVDF) membrane. After the transfer solution on the membrane was washed away, western blocking solution was added, and the mixture was slowly shaken on a shaking table and blocked at room temperature for 2 hours. The membrane was incubated with the following primary antibodies at 4°C overnight: ACE2 (1 : 500), MasR (1 : 500), NF-*κ*B (1 : 1000), and *β*-actin (1 : 500), which were then incubated with horseradish peroxidase-conjugated secondary antibodies (Goat anti-rabbit IgG, 1 : 20000 dilution) for 1.5 h, followed by the addition of washing buffer (PBST) for 10 minutes each and 3 washings in total. Corresponding protein expressions were measured using an enhanced chemiluminescence reagent kit (Thermo, No. 34094). The semiquantitative analyses of immunoblots were performed through Image *J*.

### 2.13. Immunohistochemistry

The immunoreactive expression of MasR in the lung was observed by immunohistochemistry staining. The 4-*µ*m thick paraffin sections were cut and stored at 58°C for 3 h after dewaxing in xylene. MasR (1 : 500) primary antibody was then added and incubated overnight at 4°C. Thereafter, the sections were flushed with PBS three times and incubated with secondary antibodies (1 : 20000 dilution) for 20 min. This was followed by visualization with 3,3′-diaminobenzidine (DAB) horseradish peroxidase color development kit (Zsbio, No. ZLI-9018). Immunohistochemical positive signals were stained brown in the cell membrane [21], and the optical density (OD) was analyzed using Image Pro Plus 6.0 software.

### 2.14. Statistical Analysis

The statistical description proceeded via SPSS 22.0 software for Windows. All measured values were presented as mean ± standard deviation (SD). The intergroup differences in normal distribution were performed via one-way analysis of variance (ANOVA), which was followed by the least significant difference (LSD) test for multiple comparisons. *P* < 0.05 was considered statistically significant.

## 3. Results

### 3.1. Preliminary Chemical Analysis of XQLD

**Figure 1:**
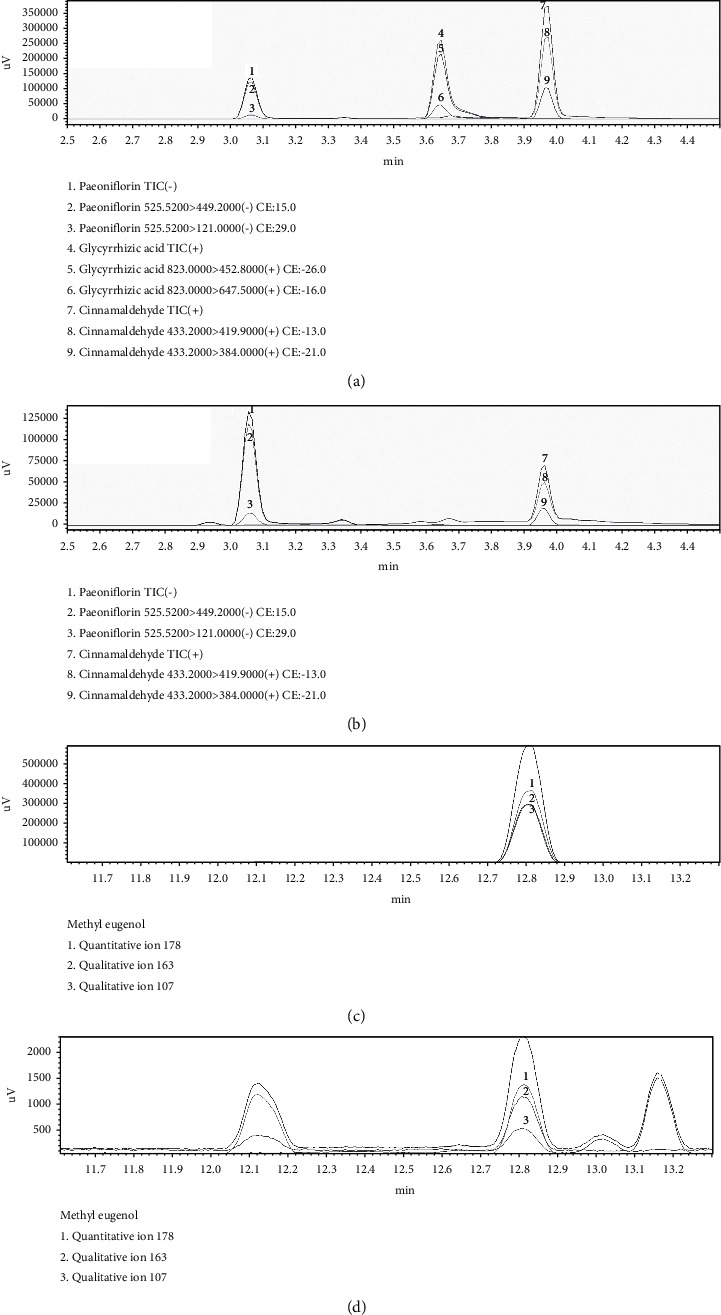
A representative chromatogram of XQLD in UPLC-MS/MS (a), (b) and GC-MS (c), (d) system. (a) The paeoniflorin, glycyrrhizic acid, and schizandrin standard; (c) the methyl eugenol standard; b and d: the sample of XQLD.

Chemical components of XQLD were preliminary analyzed using the UPLC-MS/MS and GC-MS system [9, 20]. The retention time of five marker compounds were detected at approximately 3.06 (paeoniflorin), 3.64 (glycine acid), 3.96 (Schizandrin), 10.42 (cinnamaldehyde), and 12.81 (methyl eugenol) min.  Figure 1 is the representative chromatogram and further quantified with definitive standards (Table 3).

### 3.2. XQLD Ameliorated Lung Function in Septic ALI Rats

Compared with the control group, the lung function of model rats with septic lung injury induced by LPS was seriously damaged (Table 4). Our results indicated that XQLD pretreatment could ameliorate FVC, FEV0.1, FEV0.3, FEV0.1/FVC, and FEV0.3/FVC (*P* < 0.001∼0.01) in a dose-dependent manner. XQLD of high-dose (*P* < 0.001) had a better effect than that of low-dose (*P* < 0.01) and middle-dose (*P* < 0.001∼0.01) groups, and the medium dose is superior to the low-dose group. Furthermore, both XQLD-H (*P* < 0.001) and DEX (*P* < 0.001) groups showed equally potent prophylactic effects in septic ALI rats.

The values were presented as mean ± standard deviation (SD) and the intergroup differences were performed via one-way analysis of variance followed by least significant difference (LSD) test for multiple comparisons. (*n* = 8 per group, ^▲^*P* < 0.05, ^▲▲^*P* < 0.01, ^▲▲▲^*P* < 0.001, vs control group; ^#^*P* < 0.05, ^##^*P* < 0.01, ^###^*P* < 0.001, vs ALI group; ^∆^*P* < 0.05, ^∆∆^*P* < 0.01, ^∆∆∆^*P* < 0.001, vs DEX group). FVC: forced vital capacity; FEV0.1: forced expiratory volume in 0.1 s; FEV0.3: forced expiratory volume in 0.3 s; ALI: acute lung injury; XQLD-L: Xiaoqinglong decoction low dose (6.25 mg/kg/d); XQLD-M: Xiaoqinglong decoction medium dose (12.5 mg/kg/d); XQLD-H: Xiaoqinglong decoction high dose (25 mg/kg/d); DEX: dexamethasone (1 mg/kg).

### 3.3. XQLD Improved Arterial Blood Gas in Septic ALI Rats

**Figure 2:**
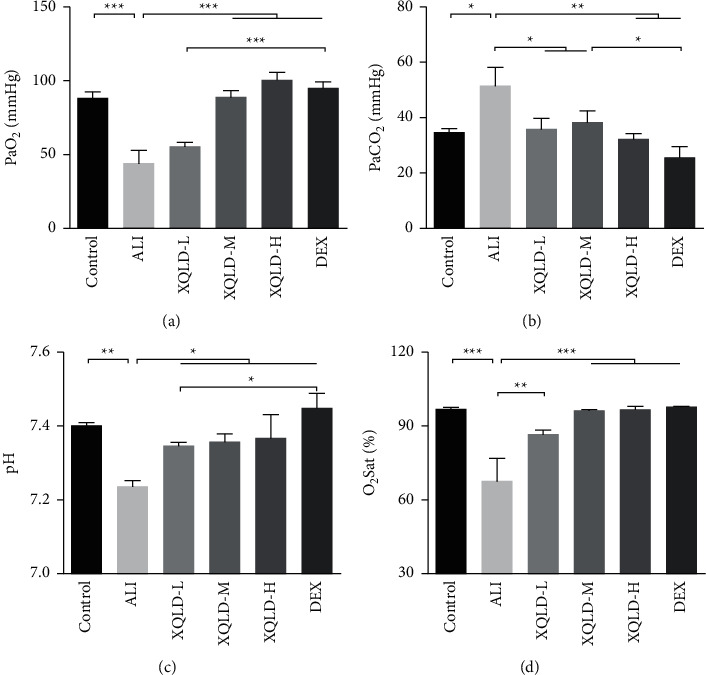
Effects of XQLD on arterial blood gas in LPS-induced septic ALI rats. PaO_2_ (a); PaCO_2_ (b); pH (c); O_2_Sat (d). The values were presented as mean ± standard deviation (SD), and the intergroup differences were performed via one-way analysis of variance followed by the least significant difference (LSD) test for multiple comparisons (*n* = 8 per group, ^*∗*^*P* < 0.05, ^*∗∗*^*P* < 0.01, ^*∗∗∗*^*P* < 0.001). ALI: acute lung injury; XQLD-L: Xiaoqinglong decoction low dose (6.25 mg/kg/d); XQLD-M: Xiaoqinglong decoction medium dose (12.5 mg/kg/d); XQLD-H: Xiaoqinglong decoction high dose (25 mg/kg/d); DEX: dexamethasone (1 mg/kg).

As depicted in Figure 2, the LPS treatment resulted in a remarkable decrease in PaO_2_ (*P* < 0.001), pH (*P* < 0.01) and O_2_Sat (*P* < 0.001) levels (Figures 2(a), 2(c) and 2(d)), and an increase in PaCO_2_ (*P* < 0.05) (Figure 2(b)), while blood gas was greatly improved in the XQLD and DEX groups (Figure 2). Furthermore, the pH value in the XQLD-L (*P* < 0.05) group was clearly more normalized than that in the DEX (*P* < 0.05) group after the LPS challenge for 24 h (Figure 2(c)). However, the amelioration of the DEX (*P* < 0.05) group on PaO_2_ (Figure 2(a)) was superior. It was noted that the XQLD-M group had a significantly superior PaCO_2_ value compared with the DEX group (*P* < 0.05) (Figure 2(b)). The measurements indicated no significant differences in O_2_Sat between the XQLD and DEX groups (Figure 2(d)).

### 3.4. XQLD Alleviated Pulmonary Impairment in Septic ALI Rats

**Figure 3:**
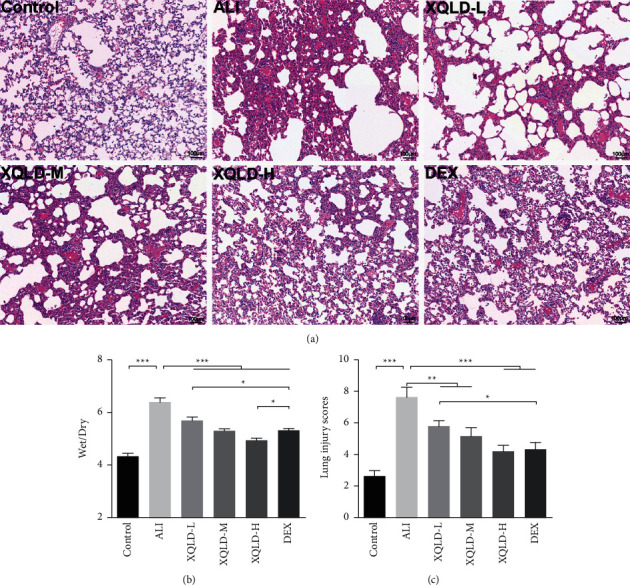
Effect of XQLD on lung histomorphology in LPS-induced septic ALI rats. Hematoxylin and eosin (H&E) (a); lung wet/dry (W/*D*) ratio (b); lung injury scores (c). The values were presented as mean ± standard deviation (SD), and the intergroup differences were performed via one-way analysis of variance followed by the least significant difference (LSD) test for multiple comparisons (*n* = 8 per group, ^*∗*^*P* < 0.05, ^*∗∗*^*P* < 0.01, ^*∗∗∗*^*P* < 0.001). ALI: acute lung injury; XQLD-L: Xiaoqinglong decoction low dose (6.25 mg/kg/d); XQLD-M: Xiaoqinglong decoction medium dose (12.5 mg/kg/d); XQLD-H: Xiaoqinglong decoction high dose (25 mg/kg/d); DEX: dexamethasone (1 mg/kg).

H&E staining showed that the lung tissues of rats were severely damaged after LPS administration for 24 h, with massive inflammatory cell infiltration in the pulmonary interstitium and alveolar cavity, edema of the parenchyma and alveolar hemorrhage compared with the control group (Figure 3(a)). In contrast, these pathological damages were alleviated by XQLD (*P* < 0.001) and DEX (*P* < 0.001) pretreatment (Figure 3(b)), the W/*D* of XQLD-H group is lower than that of the DEX group (*P* < 0.05), which indicates that the XQLD-H group has a better effect on pulmonary edema. Furthermore, the septic rats showed markedly higher lung injury scores than those of control rats (*P* < 0.001) (Figure 3(c)). Compared with the ALI group, pathological scores decreased in the XQLD (*P* < 0.01) and DEX (*P* < 0.001) rats (Figure 3(c)). Moreover, with the increase in dose, XQLD emerged better effects on relieving pulmonary injury. It is suggested that XQLD could ameliorate pulmonary impairment in LPS-induced septic ALI rats.

### 3.5. XQLD Relieved the Inflammatory Response and Oxidative Damage in Septic ALI Rats

**Figure 4:**
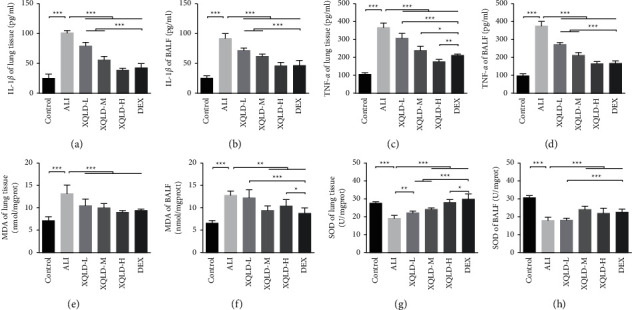
Effects of XQLD on the concentrations of IL-1*β* (a), (b) and TNF-*α* (c), (d) in lung tissue and BALF of LPS-induced septic ALI rats by ELISA, as well as the MDA (e), (f) content and SOD (g), (h) activity. The values were presented as mean ± standard deviation (SD), and the intergroup differences were performed via one-way analysis of variance followed by the least significant difference (LSD) test for multiple comparisons (*n* = 8 per group, ^*∗*^*P* < 0.05, ^*∗∗*^*P* < 0.01, ^*∗∗∗*^*P* < 0.001). ALI: acute lung injury; XQLD-L: Xiaoqinglong decoction low dose (6.25 mg/kg/d); XQLD-M: Xiaoqinglong decoction medium dose (12.5 mg/kg/d); XQLD-H: Xiaoqinglong decoction high dose (25 mg/kg/d); DEX: dexamethasone (1 mg/kg). BALF: bronchoalveolar lavage fluid. IL-1*β*: interleukin-1*β*; TNF-*α*: tumor necrosis factor-*α*; MDA: malonaldehyde; SOD: superoxide dismutase.

To determine whether XQLD could inhibit inflammatory responses and oxidative stress, the contents of IL-1*β* and TNF-*α* in rat lung tissues and BALF were examined by ELISA, moreover, the expression of MDA and SOD in the lung were also detected. As depicted in Figure 4, LPS intraperitoneal injection resulted in a drastic increase of IL-1*β* (*P* < 0.001) (Figures 4(a) and 4(b)) and TNF-*α* (*P* < 0.001) (Figures 4(c) and 4(d)) secretion in rats. Chemical detections revealed that the MDA (*P* < 0.001) level increased and SOD (*P* < 0.001) activity decreased in ALI group rats compared with the control group. Still, there were significantly improved for MDA (*P* < 0.001∼0.01) and SOD (*P* < 0.001∼0.01) after XQLD and DEX pretreatments, whether in the lung or BALF (Figures 4(e)–4(h)). These results were particularly significant in the high-dose XQLD and DEX groups. In addition, there was no marked difference in MDA levels between the XQLD and DEX groups (Figure 4(e)). The results suggested that XQLD could relieve the inflammatory response and oxidative damage in the lung of LPS-induced septic ALI rats.

### 3.6. XQLD Promoted the Expressions of ACE2 and MasR, the Level of Ang (1–7), and Inhibited the Expression of NF-Κb in Septic ALI Rats

**Figure 5:**
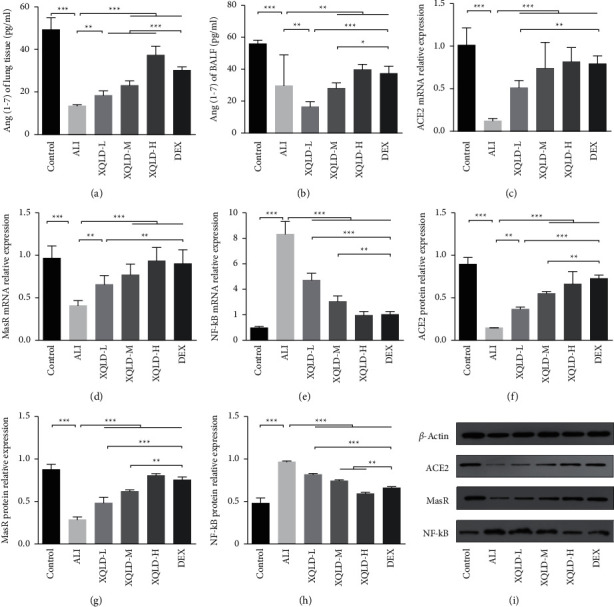
Effects of XQLD on the concentrations of Ang (1–7) in BALF and lung tissue by ELISA (a and b), as well as the mRNA and protein expression of ACE2, MasR, and NF-*κ*B in lung tissue of LPS-induced septic ALI rats by RT-qPCR (c–e) and western blotting (f–i). The values were presented as mean ± standard deviation (SD), and the intergroup differences were performed via one-way analysis of variance followed by the least significant difference (LSD) test for multiple comparisons (*n* = 8 per group, ^*∗*^*P* < 0.05, ^*∗∗*^*P* < 0.01, ^*∗∗∗*^*P* < 0.001). ALI: acute lung injury; XQLD-L: Xiaoqinglong decoction low dose (6.25 mg/kg/d); XQLD-M: Xiaoqinglong decoction medium dose (12.5 mg/kg/d); XQLD-H: Xiaoqinglong decoction high dose (25 mg/kg/d); DEX: dexamethasone (1 mg/kg). ACE2: angiotensin-converting enzyme 2; Ang (1–7): angiotensin (1–7). MasR: mitochondrial assembly receptor; NF-*κ*B: nuclear factor-*κ*B.

To investigate the protective mechanism of XQLD on lung inflammation and oxidative damage in ALI rats, its effects on the expressions of ACE2 and MasR, and the Ang (1–7) level and the expression of NF-*κ*B were observed. As depicted in Figure 5, LPS administration down-regulated the level of Ang (1–7) (*P* < 0.001) (Figures 5(a) and 5(b)) in the lung and BALF, the mRNA and protein expressions of ACE2 (*P* < 0.001) and MasR (*P* < 0.001∼0.01), while promoted the expression of NF-*κ*B (*P* < 0.001) in the lung (Figures 5(c)–5(h)). In contrast, both XQLD and DEX promoted the expressions of ACE2 (*P* < 0.001∼0.01). and MasR (*P* < 0.001), and the Ang (1–7) (*P* < 0.001∼0.01) level inhibited the expression of NF-*κ*B (*P* < 0.001) (Figure 5).

**Figure 6:**
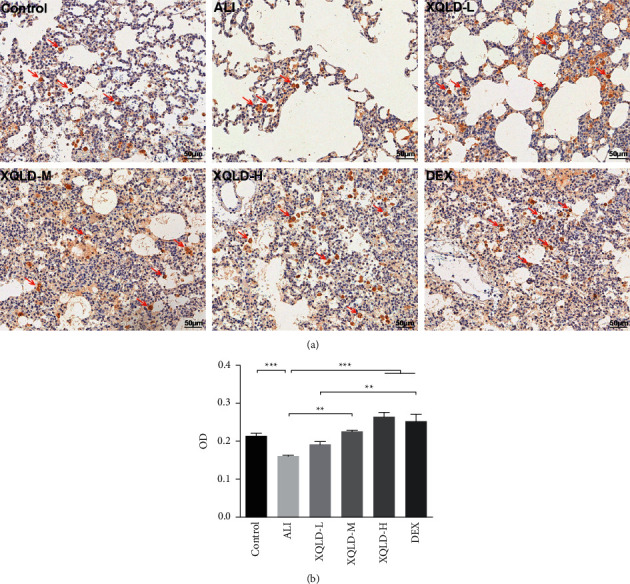
The positive expression of Mas in lung tissue (× 200 times). Immunohistochemical staining showing immunoreactive cells for MasR in the lungs of different groups (as shown by the red arrows in Figure 6. (a) and the OD were analyzed (b). The values were presented as mean ± standard deviation (SD), and the intergroup differences were performed via one-way analysis of variance followed by the least significant difference (LSD) test for multiple comparisons. (*n* = 8 per group, ^*∗*^*P* < 0.05, ^*∗∗*^*P* < 0.01, ^*∗∗∗*^*P* < 0.001). ALI: acute lung injury; XQLD-L: Xiaoqinglong decoction low dose (6.25 mg/kg/d); XQLD-M: Xiaoqinglong decoction medium dose (12.5 mg/kg/d); XQLD-H: Xiaoqinglong decoction high dose (25 mg/kg/d); DEX: dexamethasone (1 mg/kg); OD: optical density.

Furthermore, the results of immunohistochemistry revealed that the expression of MasR in lung tissue was consistent with that of RT-qPCR and western blotting (Figure 6(a)). MasR is widely distributed, mainly in bronchial epithelial cells [22], lung eosinophils [23], and lung mast cells [24]. The OD value decreased significantly in theALI group rats, while ameliorated in a dose-dependent manner in the XQLD pretreatment group rats (Figure 6(b)).

### 3.7. Pearson Correlation

**Figure 7:**
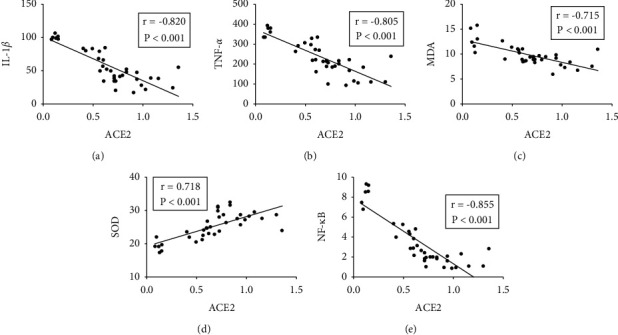
Pearson correlation between mRNA expression of ACE2 and contents of IL-1*β*, TNF-*α*, MDA, SOD, and NF-*κ*B in lung tissue.

Pearson correlations were carried out between mRNA expressions of ACE2 and contents of IL-1*β*, TNF-*α*, MDA, SOD, and expression of NF-*κ*B in lung tissue. The results showed that the expressions of ACE2 were inversely related to IL-1*β*, TNF-*α*, MDA, and NF-*κ*B (Figures 7(a)–7(c) and 7(e)) and positively correlated with SOD contents (Figure 7(d)).

## 4. Discussion

Sepsis has long been considered a major health problem worldwide with high mortality rates, of which the ALI and ARDS increase the risk of multiple organ failures as major complications in severe sepsis [25]. As a common pathogenic factor associated with ALI, LPS mainly exists in the cell wall of Gram-negative bacteria [26]. The major pathologies related to ALI are inflammatory infiltration, pulmonary edema, and pulmonary microvascular injury caused by inflammatory factors [27]. The massive exocytosis of proinflammatory cytokines and the development of protein edema areas aggravate the pulmonary infection cascade [28]. As a Chinese traditional prescription, XQLD has been widely used clinically for the therapeutics of respiratory diseases, including bronchial asthma, allergic rhinitis [29], and chronic obstructive pulmonary disease [30]. As shown by UPLC-MS/MS (Table 3 and Figure 1(a)), paeoniflorin is the main component of paeoniae radix in XQLD [9, 10, 19], which protects against lung injury by alleviating oxidative stress [31] and inflammatory cell infiltration [32]. In this research, we explored the preventive effect of XQLD on septic ALI induced by LPS and its possible mechanisms.

In sepsis, the lung is the most vulnerable target organ [4]. The major clinical characteristics of ALI are respiratory distress, hypoxemia, extensive capillary exudation, and noncardiogenic pulmonary edema [33, 34]. In our study, XQLD and DEX significantly improved lung function in ALI rats. Moreover, H&E staining and wet/dry (W/*D*) measurement results showed that XQLD and DEX significantly reduced inflammatory infiltration and edema of lung tissue in septic ALI rats. Lung injury usually leads to diminished oxygenation and further aggravates the systemic inflammatory response [35]. Our results demonstrated that XQLD significantly improved arterial blood gas, increased blood oxygen saturation, and improved pH, which was comparable to that of DEX.

The signaling molecules IL-1*β* and TNF-*α* exert a vital deteriorating effect on the systemic inflammatory response. The severity of lung injury has been demonstrated to correlate with IL-1*β* and TNF-*α* activity [36]. Furthermore, MDA is a lipid peroxidation product mediated by ROS, which is an alternative marker of oxidative stress. SOD presents in lung tissue abundantly and protects the lung tissue from ALI by scavenging ROS [37]. Our date results exhibited that XQLD significantly attenuated IL-1*β*, TNF-*α*, and MDA levels, increased SOD activity in lung tissue and BALF, which suggested that the preventive effects of XQLD are brought by mediating the depression of inflammatory response, restoring the defensive activity of endogenous antioxidants, and reducing the lipid peroxidation levels.

As a pivotal transcription factor, NF-*κ*B directly modulates the expression of inflammatory cytokines such as IL-1*β*, TNF-*α*, and IL-6 [38]. In general, oxidative stress is related to inflammatory responses. Excessive production of ROS also triggers oxidative damage, which alters the endogenous antioxidant defense system, such as the decrease in SOD activity [39]. In this regard, studies have shown that ROS can activate the NF-*κ*B pathway, thus raising inflammatory mediators [40], which suggests that the NF-*κ*B activity may be involved in the oxidative stress process and the subsequent pathological process of lung injury. The present findings demonstrated that the NF-*κ*B activity was significantly raised in ALI rats, whereas both pretreatments of XQLD and DEX showed a great restriction for the NF-*κ*B activity. These results indicated that XQLD alleviated the inflammatory reaction, and oxidative damage may be related to the down-regulation of NF-*κ*B.

Anti-inflammatory characteristics of XQLD are not only related to the inhibition of *κ*B*α* (I*κ*B*α*)/NF-*κ*B signaling but other signaling such as mitogen-activated protein kinases (MAPKs), which produce pro-inflammatory mediators and chemokines to regulate the inflammatory response [41]. It has been reported that XQLD can upregulate the expression of MAPKs [10, 42]. In an in vitro study, the anti-inflammatory activity of XQLD may be mediated by suppressing the activation of NF-*κ*B and phosphorylation of c-Jun NH2-terminal kinases (JNK) and ERK1/2 signals in LPS-stimulated RAW 264.7 cells [11].

In terms of our results, the protein and mRNA relative expressions of ACE2 and MasR, as well as Ang (1–7) levels in the lung of ALI rats were all suppressed. In contrast, NF-kB levels increased significantly, suggesting that LPS administration could trigger severe inflammation in the lung, accompanied by down-regulation of upstream signals like ACE2 and up-regulation of NF-kB activity. Moreover, the lung tissues of ALI rats pretreated with XQLD and DEX were protected against LPS infection, in which the expression of ACE2 was activated while the expression of NF-kB was inhibited. Furthermore, Pearson correlation showed that ACE2 expressions were inverse to NF-kB, IL-1*β*, TNF-*α*, and MDA, while positively correlated with SOD contents. These results suggested that XQLD may reduce the expression of downstream NF-kB and inflammatory factors, as well as lipid peroxidation damage by promoting the expression of ACE2. Therefore, we speculated that RAS may be an important new therapeutic target for septic ALI, and XQLD probably plays a certain regulatory role.

The RAS plays a vital function in modulating electrolyte balance and blood pressure [12]. Lately, it has been revealed that RAS performs vital functions in the inflammatory responses of the cardiovascular system, kidney, liver, and lung [43]. As a major member of the RAS, the ACE2-Ang (1–7)-Mas axis has been pointed to have the effects against lung fibrosis [44], pulmonary hypertension [45], pyroptosis [46], and prevents apoptosis of pulmonary microvascular endothelial cells [47], which is considered that it occurs mainly through anti-inflammatory and antioxidant pathways [12]. Renin generates the production of AngI, which translates into AngII through ACE. Contrary to ACE, ACE2 counteracts the activity of ACE as a homologue by inducing AngII to degrade into Ang (1–7), which combines with MasR and neutralizes the action of AngII [48]. Furthermore, the ACE2-Ang (1–7)-Mas axis attenuates the AngII-dependent formation of ROS in the cell nuclei, exerting a distinct defense mechanism against cell damage and oxidative stress [49]. *In vitro*, recent cell culture experiments proved that A779 (a selective MasR inhibitor) pretreatment significantly enhanced the phosphorylation of NF-kB p65 [44]. In addition, several studies confirmed that the ACE2-Ang (1–7)-Mas axis may restrain the NF-kB signaling pathway, attenuate the inflammatory reaction, and repress the generation of ROS [44, 47, 50], which provide a basis for further experiments.

## 5. Conclusion

In conclusion, current research indicated that inflammatory response and oxidative stress played a crucial role in septic ALI. In addition, XQLD actively protected rats against LPS-induced septic ALI by anti-inflammatory and antioxidants, which are possibly related to the up-regulation of the ACE2-Ang (1–7)-Mas axis and inhibition of the NF-*κ*B pathway.

## Figures and Tables

**Table 1:** Components and doses of herbal medicine in one prescription of XQLD for human.

Chinese name	Botanic family	Botanical nomenclature	Pharmacognostic nomenclature	Medicinal part	Amount (g)
Mahuang	Ephedraceae	Ephedra sinica Stapf	Ephedrae Herba	Stem	9
Guizhi	Lauraceae	Cinnamomum cassia Blume	Cinnamonomi Cortex	Twig	9
Ganjiang	Zingiberaceae	Zingiber offificinale Roscoe	Zingiberis Rhizome	Rhizome	6
Xixin	Aristolochiaceae	Asarum heterotropides F. Maekawa	Asari herba cum Radice	Whole plant	6
Wuweizi	Schisandraceae	Schisandra chinensis Baill	Schisandrae Fructus	Fruit	6
Baishao	Ranunculaceae	Paeonia lactiflflora Pall	Paeoniae Radix	Root	9
Banxia	Araceae	Pinellia ternate Breitenbach	Pinelliae Tuber	Tuber	9
Gancao	Leguminosae	*Glycyrrhiza* uralensis Fisch.	Glycyrrhizae Radix	Root	6

**Table 2:** Primer sequences of ACE2, MasR, NF-*κ*B, and *β*-actin.

Gene	Forward primer	Reverse primer	Product
ACE2	5′-ATGAAGAGTATGTGGTCCTG-3′	5′-AGAGTTTCGGTTCACTCGGC-3′	124 bp
MasR	5′-GGCTTTCTGGATTCTCAA-3′	5′-TTCCCTTCCTGTTTCTCA-3′	143 bp
NF-*κ*B	5′-ACCCCTTCCAAGAAGAGCAG-3′	5′-AGAGTTTCGGTTCACTCGGC-3′	185 bp
*β*-actin	5′-CCCATCTATGAGGGTTACGC-3′	5′-TTTAATGTCACGCACGATTTC-3′	150 bp

ACE2: angiotensin-converting enzyme 2; MasR: mitochondrial assembly receptor; NF-*κ*B: nuclear factor-*κ*B.

**Table 3:** Herbal pharmacognostic sources and contents of main ingredients in XQLD.

Ingredient	Herbal source	Ingredient/XQLD (*µ*g/g)
Paeoniflorin	*Paeoniae Radix*	519.265
Glycyrrhizic acid	*Glycyrrhizae Radix*	25.319
Schizandrin	*Schisandrae Fructus*	7.806
Cinnamaldehyde	*Cinnamonomi Cortex*	0.075
Methyl eugenol	*Asari herba cum Radice*	0.005

**Table 4:** Comparison of lung function in different groups of rats.

Group	FVC (ml)	FEV0.1 (ml)	FEV0.3 (ml)	FEV0.1/FVC (%)	FEV0.3/FVC (%)
Control	8.86 ± 0.84	2.69 ± 0.11	8.43 ± 0.69	30.30 ± 1.82	95.09 ± 1.40
ALI	7.15 ± 0.66^▲▲▲^	1.87 ± 0.09^▲▲▲^	6.13 ± 0.38^▲▲▲^	24.13 ± 1.47^▲▲▲^	79.10 ± 1.45^▲▲▲^
XQLD-L	7.83 ± 0.38^##∆∆^	2.13 ± 0.14^##∆∆^	6.83 ± 0.68^##∆∆^	27.20 ± 0.58^##∆^	87.22 ± 0.48^##∆^
XQLD-M	8.52 ± 1.04^###∆^	2.44 ± 0.05^###∆^	7.72 ± 0.14^##∆^	28.64 ± 3.16^##^	90.61 ± 2.91^##^
XQLD-H	8.98 ± 0.62^###^	2.61 ± 0.12^###^	8.31 ± 0.57^###^	29.06 ± 1.22^###^	92.54 ± 2.67^###^
DEX	9.18 ± 0.17^###^	2.69 ± 0.16^###^	8.38 ± 0.14^###^	29.30 ± 2.03^###^	91.29 ± 2.11^###^

## Data Availability

Data will be available from the authors upon reasonable request.
